# Coronary Artery Disease: A Key Issue in Type 2 Myocardial Infarction: Systematic Review and Recent Findings

**DOI:** 10.3390/jcm12196412

**Published:** 2023-10-09

**Authors:** Hermann Yao, Marianne Zeller, Roland N’Guetta, Yves Cottin, Alain Putot

**Affiliations:** 1 PEC2, EA 7460, UFR Health Sciences, University of Bourgogne Franche Comté, 25000 Dijon, France; marianne.zeller@u-bourgogne.fr; 2 Interventional Cardiology Department, Abidjan Heart Institute, Abidjan 01 BP V 206, Côte d’Ivoire; rolandnguetta@hotmail.com; 3 Cardiology Department, University Hospital Center Dijon Bourgogne, 21000 Dijon, France; yves.cottin@chu-dijon.fr; 4 Internal Medicine Department, Mont Blanc Hospital, 74703 Sallanches, France; a.putot@ch-sallanches-chamonix.fr

**Keywords:** type 2 myocardial infarction, coronary angiography, coronary artery disease, prognosis, secondary prevention medications

## Abstract

Underlying coronary artery disease (CAD) is increasingly considered to be a key issue in the pathophysiology of type 2 myocardial infarction (T2MI). In T2MI, which is attributable to a mismatch between oxygen supply/demand, CAD is common and appears to be more severe than in type 1 myocardial infarction (T1MI). Little is known about the heterogeneous mechanisms that cause supply/demand imbalance and non-coronary triggers leading to myocardial ischemia or about how they are potentially modulated by the presence and severity of CAD. CAD seems to be underrecognized and undertreated in T2MI, even though previous studies have demonstrated both the short and long-term prognostic value of CAD in T2MI. In this literature review, we attempt to address the prevalence and severity of CAD, challenges in the discrimination between T2MI and T1MI in the presence of CAD, and the prognostic value of CAD among patients with T2MI.

## 1. Introduction

Type 2 myocardial infarction (T2MI) remains an enigmatic clinical entity, and there are still many uncertainties regarding diagnostic and prognostic criteria, biomarkers for ischemia, the ischemic threshold, the role of cardiac imaging, and management strategies [1]. Although considered to be a cornerstone of prognosis and targeted treatment strategies, underlying coronary artery disease (CAD) is often unrecognized and undertreated in T2MI [2]. Thus, a better understanding of this ambiguous entity, and especially the role of CAD, is of particular interest [2]. The clinical classification of T2MI was introduced in 2007 [3] and redefined in 2012 [4]. The most recent revision was for the fourth universal definition of MI (UDMI) in 2018 [5]. A diagnosis of T2MI is based on criteria for myocardial infarction (MI), with evidence of an imbalance between myocardial oxygen supply and demand and in the absence of atherothrombosis. T2MI is the consequence of three interrelated factors: (1) vascular aging, including atherosclerosis; (2) predisposing chronic cardiac and extra-cardiac conditions and (3) ischemic triggers [5,6]. A wide range of conditions often encountered in the management of geriatric patients can lead to T2MI [4,7,8,9]. In 2019, de Lemos proposed a redefinition of T2MI and Type 1 MI (T1MI) derived from the fourth UDMI, considering both the underlying pathophysiological mechanisms and the management of T2MI [10]. The authors proposed that some subtypes based on pathophysiological mechanisms and management move from T2MI toward T1MI. These subtypes include spontaneous coronary artery dissection, coronary embolism, and vasomotor abnormalities. Moreover, in this proposal, CAD was the core of the clinical issue, as T2MI was categorized into two sub-groups based on the presence/lack of obstructive CAD, with ≥ 50% stenosis in at least one coronary vessel [10]. There are major therapeutic implications related to the subclassification of T2MI according to the presence or absence of significant CAD because subsequent management approaches differ substantially. Moreover, although prognostic studies on CAD in T2MI are scarce, they suggest a potent predictive value [11,12].

Based on a systematic review of the literature, we aimed to describe the prevalence, diagnosis, prognosis, and management of CAD in T2MI. 

## 2. Materials and Methods

**Figure 1:**
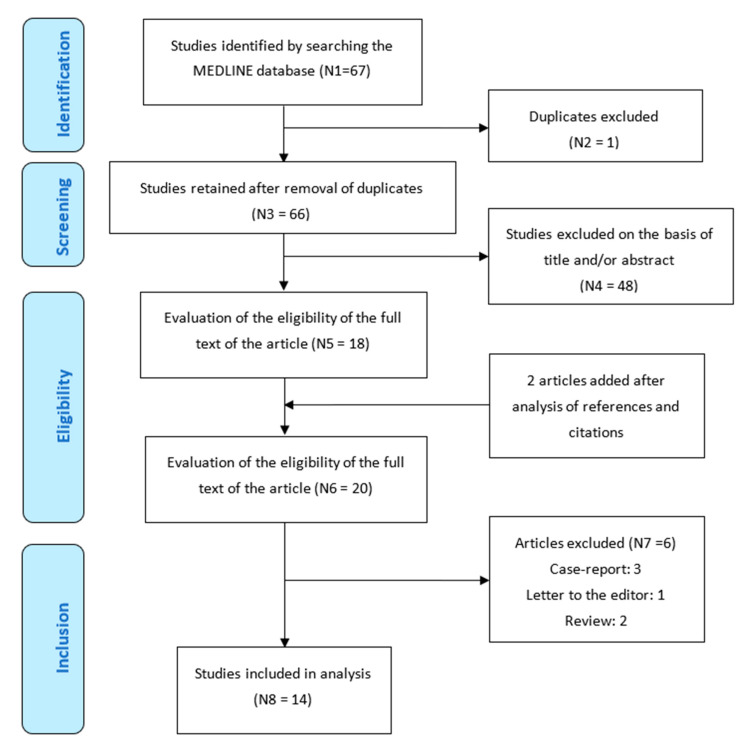
Flow chart of the study.

For this review, we identified all English language studies assessing CAD in T2MI patients published between 2012 and 2022 using the MEDLINE database (PubMed; National Library of Medicine, NIH, Bethesda, MD, USA) in accordance with PRISMA standards [13] (Figure 1). The studies are summarized in Table 1 [8,11,12,14,15,16,17,18,19,20,21,22,23,24].

## 3. T2MI Criteria for Diagnosis

Acute MI is currently defined based on the fourth UDMI [5]. T2MI was defined as MI secondary to myocardial oxygen supply/demand imbalance and triggered by an acute stressor. These conditions are defined in Table 2 [4,7,8,9,25].

## 4. Prevalence and Severity of CAD in T2MI Based on Coronary Angiography

**Figure 2:**
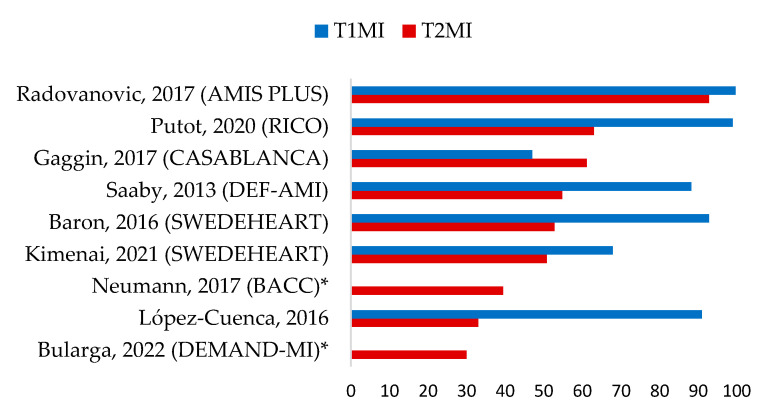
Prevalence of significant coronary artery disease (≥50%) according to type 1 and type 2 myocardial infarction patients undergoing coronary angiography (%) [8,12,14,16,19,20,21,22,24]; T1MI: type 1 myocardial infarction. T2MI: type 2 myocardial infarction. AMIS PLUS: national Registry of Acute Myocardial Infarction in Switzerland; CASABLANCA: Catheter Sampled Blood Archive in Cardiovascular Diseases; DEF-AMI: consequences of the universal 2007 Definition of Acute Myocardial Infarction studied in a Danish consecutive hospital population; SWEDEHEART: Swedish Web-system for Enhancement and Development of Evidence-based care in Heart disease Evaluated According to Recommended Therapies; BACC: Biomarkers in Acute Cardiac Care; DEMAND-MI: Determining the Mechanism of Myocardial Injury and Role of Coronary Disease in Type 2 Myocardial Infarction; * non applicable for T1MI.

CAD has been presented as an important determinant in T2MI categorization. Moreover, fixed stable coronary atherosclerosis, as a promoter of myocardial oxygen balance mismatch, is a key factor in T2MI pathophysiology [5]. Obstructive atherosclerosis is a common condition in T2MI patients who undergo coronary angiography, with a prevalence ranging from 30% to 68% [14] (Figure 2). In the nationwide Swedish Web-system for Enhancement and Development of Evidence-based care in Heart disease Evaluated According to Recommended Therapies (SWEDHEART) registry, CAD accounted for 57.6% of cases among patients with T2MI [12]. In the Catheter Sampled Blood Archive in Cardiovascular Diseases (CASABLANCA) study [19], coronary angiography was performed in 152 included T2MI patients, of whom 61.2% had two-vessel disease (≥50% stenosis). 

However, variations in the prevalence of CAD partly depend on the rates of patients who undergo coronary angiography. A recent meta-analysis by White et al [26] included 40 cohort studies with 13,803 T2MI patients and 98,930 T1MI patients. T2MI patients were less likely to undergo coronary angiography (OR 0.09; 95%CI 0.06–0.12), leading to an underestimation of the true prevalence of CAD. When compared with T1MI, T2MI patients are usually older, with higher rates of comorbidities, so angiographic explorations tend to be less frequent. In a large multicenter randomized clinical trial testing the implementation of a high-sensitivity-cardiac Troponin I (hs-cTn I) assay and UDMI recommendations in consecutive MI patients in Scotland, only 10% of T2MI patients underwent angiography compared to 59% of T1MI patients [27]. In a cross-sectional study from the 2018 National Inpatient Sample in the USA, including 268,850 patients admitted for T2MI, only 11.2% of T2MI were managed invasively, of which only 17.9% underwent coronary revascularization. There are wide disparities in the rate of coronary angiography use, mainly related to insurance status and geographic regions and independent of patient and hospital factors [28]. When compared with conservative management and after propensity-matched analyses, invasive management reduced in-hospital mortality by 30% in T2MI patients (OR 0.70; 95%CI, 0.59–0.84) [28].

Moreover, thresholds used for identifying obstructive CAD during angiography vary across studies, thus influencing the rate of patients with CAD. In a recent meta-analysis, obstructive CAD was found in 34% of T2MI cases, with the definition of coronary vessel narrowing varying between 50 and 70% [26]. In the CASABLANCA study, almost half of T2MI patients (47.7%) had ≥70% stenosis in at least two vessels, and 61.2% had ≥50% stenosis [19]. Among T2MI patients, the presence and severity of CAD can also differ according to sex, as women are less likely to have obstructive CAD than men (34% vs. 67%, respectively) [16].

Another key issue is that underlying CAD has been shown to be more severe in T2MI than in T1MI [17,19,23]. In patients admitted to the emergency department (ED) with a history of CAD, those with T2MI are more likely to have left main or three-vessel disease (with ≥ 50% stenosis) (56% of T2MI vs. 43% of T1MI, *p* = 0.015) [17]. In the CASABLANCA study, a higher CAD burden in T2MI was observed no matter the threshold used to define significant stenosis. Compared to T1MI, the rate of three-vessel disease in T2MI was higher for all thresholds, i.e., ≥30% stenosis (57.4% vs. 42.8%, *p* = 0.002), ≥50% stenosis (41.1% vs. 27.1%, *p* = 0.001), and ≥70% stenosis (25.0% vs. 16.6%, *p* = 0.02) [19].

In most studies, classical CV risk factors are frequently associated with T2MI and CAD [29]. Beyond these classical risk factors, non-conventional risk factors such as depression can also contribute to the pathogenesis of CAD, particularly in T2MI [30].

## 5. Diagnostic Methods for Detecting CAD in T2MI

Coronary angiography remains the gold standard technique for detecting CAD in both T1MI and T2MI. Although T2MI diagnosis is based on myocardial oxygen mismatch in the absence of atherothrombosis, plaque disruption or intracoronary thrombus are not exclusive to T1MI, leading to confusion regarding the diagnostic criteria to be considered [31]. Indeed, coronary angiography and intravascular ultrasound imaging (IVUS) findings showed plaque rupture in patients with MI (33% of cases) as well as in stable (11%) and asymptomatic CAD patients (11%) [32]. An ongoing randomized trial is expected to provide more comprehensive data on the appropriateness of coronary investigations in patients with T2MI [33]. In addition to coronary angiography, cardiac magnetic resonance (CMR) imaging could help discriminate between MI types. In a study among 100 patients with T2MI, systematic coronary and CMR imaging led to the reclassification of seven patients to T1MI and myocardial injury [14]. It is important to note that differentiating between T1MI, T2MI, and even myocardial injury is often a clinical challenge because of the various differential diagnoses associated with cTn elevation, potentially leading to the misdiagnosis of T2MI [34]. The diagnostic and prognostic value of CMR is of particular interest in patients with a working diagnosis of myocardial infarction with non-obstructive coronary arteries (MINOCA) [35,36]. Both a CMR-confirmed diagnosis of MINOCA and a myocardial extension of late gadolinium enhancement are associated with an increased risk of major adverse cardiovascular events at follow-up [35,36]. Coronary computed tomography angiography (CCTA), often used as a rule-out strategy in patients with inconclusive tests, helps identify the presence of obstructive or non-obstructive plaque and guides preventative medical therapies [37]. In patients with normal coronary arteries, non-obstructive coronary disease, or distal obstructive disease, invasive imaging is not required [38]. Finally, stress echocardiography and global longitudinal strain on resting echocardiography may sometimes be useful to identify CAD [39].

## 6. History of CAD in T2MI and Clinical Implications

**Figure 3:**
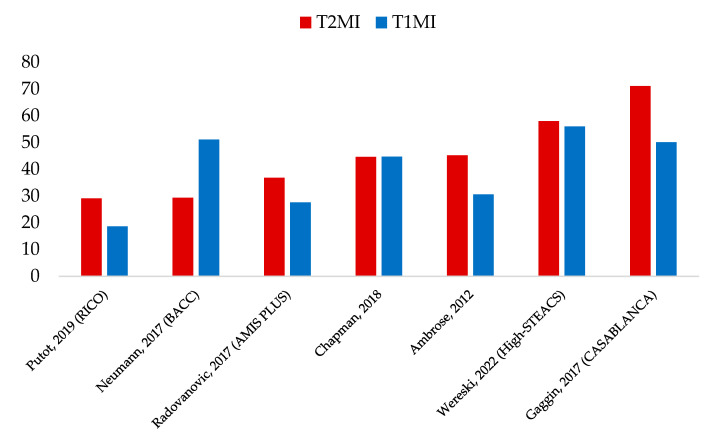
Prevalence of history of coronary artery disease according to type 1 and type 2 myocardial infarction (%) [11,15,17,19,20,21,23]; T1MI: type 1 myocardial infarction; T2MI: type 2 myocardial infarction; RICO: ObseRvatoire des Infarctus de la Côte d’Or; BACC: Biomarkers in Acute Cardiac Care; RICO: obseRvatoire des Infarctus de la Côte d’Or; AMIS PLUS: national Registry of Acute Myocardial Infarction in Switzerland; High-STEACS: High-Sensitivity Troponin in the Evaluation of patients with Acute Coronary Syndrome; CASABLANCA: Catheter Sampled Blood Archive in Cardiovascular Diseases.

A history of CAD, i.e., medically documented CAD, is another way to address CAD. T2MI patients frequently have a history of CAD, apparently more often than T1MI patients, with rates beyond 70% in the CASABLANCA study [19,24,40] (Figure 3). However, recent data from the High-STEACS (High-Sensitivity Troponin in the Evaluation of Patients with Suspected Acute Coronary Syndrome) study suggested similar rates of known CAD among patients with T1MI or T2MI (56% vs. 58%, respectively) [15]. In patients admitted to the ED, CAD history was an independent predictive factor for T2MI versus T1MI, increasing the risk by almost 40% (OR 1.38; 95%CI 1.08–1.77) [17]. Finally, the history of CAD in T2MI has an important CV prognostic value because it increases the risk for both T1MI (aHR 1.34, 95%CI 1.28–1.42) and T2MI (aHR 1.11, 95%CI 1.07–1.16) at one year of follow-up [15].

## 7. Discrimination of T2MI vs. T1MI in the Presence of CAD: Role of Biomarkers

In the absence of specific biomarkers, differentiation between T1MI and T2MI is based on clinical criteria [5], but it can be difficult to make a differential diagnosis in some situations. Surprisingly, in a relatively small sample of T2MI patients, Bularga et al. showed a slightly higher peak of hs-cTn I in patients without CAD vs. with CAD on cardiac imaging (magnetic resonance or echocardiography) [14]. When comparing patients with previous CAD in T1MI and T2MI, T2MI had higher CRP levels, whereas T1MI patients had a cTn peak approximately eight times higher; the CRP/Tn I ratio had the best predictive values with an area under the curve (AUC) of 0.84 (95%CI: 0.81–0.87) to discriminate between T2MI and T1MI [17].

Interestingly, underlying CAD in T2MI patients has been associated with preferential triggering mechanisms. Tachyarrhythmia and acute anemia/bleeding were more likely to occur in T2MI with CAD, while respiratory insufficiency was more prevalent in T2MI without CAD [12]. Further studies are needed to address this gap in knowledge on the underlying pathophysiology of T2MI and to elucidate how CAD burden could promote acute myocardial oxygen balance mismatch in patients with tachyarrhythmia or acute anemia related to severe bleeding.

In T2MI, the myocardial oxygen supply/demand imbalance attributable to acute myocardial ischemia is often multifactorial. It can be related to reduced myocardial perfusion due to fixed coronary atherosclerosis without plaque rupture in large vessel stenosis and to coronary microvascular dysfunction, including endothelial dysfunction, smooth muscle cell dysfunction, and sympathetic innervation dysregulation [5]. Coronary microvascular dysfunction is a major cause of myocardial ischemia, is associated with a high risk of poor outcomes, and often associated with CV risk factors such as diabetes [41]. Furthermore, there is a close interaction between microvascular and epicardial CAD, and coronary microvascular dysfunction has been shown to be a strong prognostic factor for patients with and without significant stenosis [42]. Moreover, it has been suggested that microcirculatory dysfunction can be a confounder of CAD in evaluating the hemodynamic status of coronary circulation [43].

## 8. Prognosis in T2MI Patients with CAD and Treatment Strategies

When compared with T1MI, T2MI is associated with a high rate of mortality, which was found to be more than two-thirds over 5 years, mostly from non-CV causes [11]. However, only a few studies have addressed the prognostic value of CAD among patients with T2MI (Table 2). Among T1MI and T2MI patients with significant CAD (defined as coronary stenosis ≥ 50%), in-hospital all-cause mortality risk was increased twofold in T2MI patients (15% vs. 7% for T1MI, *p* < 0.001). However, CV deaths were comparable between the groups [17]. CAD has been shown to have a deleterious long-term impact on T2MI. Among the 41,817 patients with T2MI and T1MI included in the SWEDHEART registry who underwent coronary angiography, T2MI patients with obstructive CAD had higher crude long-term (1.9 years) all-cause mortality than T1MI patients with obstructive CAD (HR 1.72; 95%CI 1.45–2.03) [12] (Table 3). However, after adjustments for confounders, long-term mortality risk was 30% lower in T2MI with obstructive CAD, indicating that factors other than the myocardial infarction itself influence the outcome. It should be noted, however, that coronary angiography was performed in less than one-third of patients, thus limiting the interpretation of these findings. The evidence for the prognostic impact of CAD in T2MI appears to be weak. Previous studies, including patients with MI (without distinction of clinical MI type), found that patients without significant CAD had a better short- and long-term prognosis than patients with obstructive CAD [44]. In a single-center Scottish cohort of 2122 consecutive patients with elevated cTn [11], a history of CAD was the strongest predictor of major cardiac events after 5 years in patients with T2MI (or myocardial injury) (HR 1.71; 95%CI 1.31–2.24).

Strikingly, when compared with T1MI patients with CAD, patients with T2MI and CAD are dramatically less likely to receive all secondary prevention therapies at discharge, including aspirin (66.2% vs. 90.7%), statins (69.2% vs. 86.0%) or angiotensin-converting enzyme inhibitor (52.9% vs. 71.3%, *p* < 0.001 for all). Moreover, a recent meta-analysis highlighted the lower rate of conventional cardioprotective medications in patients with T2MI (vs. T1MI), including beta blockers (58.3% vs. 76.3%), antiplatelet agents (70.8% vs. 88.5%) and statins (52.9% vs. 87.6%) [26]. 

The importance of ischemic heart disease as a component of T2MI prognosis was also recently emphasized by the development of new risk scores, such as the T2-risk score [45]. Higher T2-risk scores are associated with the occurrence of subsequent MI or all-cause death at 1 year, resulting in good performance in the derivation cohort (AUC: 0.77; 95%CI: 0.73–0.79), and both the single-center (AUC: 0.83 [95%CI: 0.77–0.88] and multicenter validation cohorts (AUC: 0.74 [95%CI: 0.64–0.83]) [45].

Finally, revascularization procedures were significantly less likely to be performed in patients with T2MI [26]. To date, although CAD in T2MI is common and associated with worse prognosis, no randomized controlled trials have evaluated invasive procedures and secondary prevention medications in this specific population, and there are no recommendations for a risk assessment or treatment strategy.

## 9. Conclusions

The underlying pathophysiology of T2MI is characterized by heterogeneous conditions leading to a mismatch between myocardial oxygen supply/demand, in association with older age and comorbidities. In addition to these underlying ischemic triggers, CAD is a frequent and severe condition in patients admitted for T2MI, in whom invasive strategies and secondary preventive medications are underused. Recent findings indicate that CAD has a strong, deleterious long-term predictive value in T2MI. Identifying underlying CAD may, thus, improve risk stratification in T2MI patients and provide a rationale for the future development of preventive therapies to reduce the risk of recurrent CV events. Machine learning models using multimodal data (patient information and ECG) have been suggested to improve MI detection [46], and integrative models, including medical data such as CAD, could thus potentially help to accurately classify T2MI and T1MI in the future.

## Figures and Tables

**Table 1:** Type 2 myocardial infarction and CAD.

Author (Year) (Study Name)	Country	Type of Study	Objectives	T2MI Patients	Results	Conclusion
Bularga (2022) [14] (DEMAND MI)	Scotland	Prospective study	Prevalence of CAD	93 patients	CAD 68% Obstructive CAD 30%	Unrecognized and untreated CAD is seen in most patients with T2MI
Wereski (2022) [15] (High-STEACS)	Scotland	Multicenter randomized trial	Risk factors for T1MI and T2MI	407 patients	Known CAD is predictor for both T1MI and T2MI	Risk factors for CAD associated with T1MI are also important predictors of T2MI events
Kimenai (2021) [16] (SWEDEHEART)	Sweden	Prospective registry	Sex differences in characteristics and outcomes in patients with T2MI	6485 patients	Obstructive CAD: 34% (women) vs. 67% (men) History of CAD was associated with all-cause death in women (HR 1.26; 95%CI 1.08–1.47)	Women with T2MI are less likely to have obstructive CAD than men Need for a sex-specific approach management of T2MI
Putot (2020) [24] (RICO)	France	Prospective registry	Characteristics and underlying causes of T2MI	862 patients	Obstructive CAD: 63% of T2MI CAD was not associated with T2MI vs. T1MI (HR 1.03; 95%CI 0.96–1.12)	Although frequent among T2MI patients, CAD was not associated with T2MI diagnosis in multivariate analysis
Putot (2019) [17] (RICO)	France	Prospective registry	CAD as a predictive factor for distinguishing T2MI/T1MI	873 patients	History of CAD 29%	Previous CAD was associated with a 40% higher risk of T2MI
Putot (2018) [18] (RICO)	France	Cross-sectional study	Predictors for in-hospital all-cause and cardiovascular mortality	947 patients	CAD is not associated with in-hospital mortality among T2MI patients	Independent predictors for CV mortality: age > 75 years, initial hemodynamic parameters, and troponin level rate at admission Predictors for all-cause mortality: initial hemodynamic parameters, creatinine, troponin, and CRP
Chapman (2018) [11]	Scotland	Prospective study	Predictor for poor outcomes among patients with T2MI or myocardial injury	429 patients	CAD was an independent predictor for MACE in patients with T2MI or myocardial injury (HR, 1.71; 95%CI 1.31–2.24).	Identification of CAD in T2MI patients may help target therapies that could modify future risks.
Gaggin (2017) [19] (CASABLANCA)	USA	Single-center prospective study	Incident T2MI	152 patients	History of CAD 71% ≥50% 2-vessel CAD 61.2% ≥70% 2-vessel CAD 47.7%	The history of CAD is a predictor for the first T2MI
Neumann (2017) [20] (BACC)	Germany	Prospective	Discrimination of patients with T2MI	99 patients	History of CAD 29.3% Obstructive CAD 39.5% 3-vessel CAD 21.1%	CAD was not a strong predictor to discriminate T1MI and T2MI
Baron (2016) [12] (SWEDEHEART)	Sweden	Prospective registry	Characteristics and long-term prognosis in T2MI and T1MI patients with and without obstructive CAD	1316 patients	T2MI with CAD: 52.8% Higher crude long-term mortality in T2MI with CAD (HR 1.72; 95%CI 1.45–2.03)	Evaluation of coronary artery status seems to have a key role in the choice of treatment and risk prediction.
Radovanovic (2016) [21] (AMIS PLUS)	Switzerland	Prospective	Incidence, presentation, treatment, and outcome of T2MI	1091 patients	History of CAD 36.8% Obstructive CAD 92.8%	The difference in the prevalence of obstructive CAD across studies is probably due to the different definitions of angiographic findings.
López-Cuenca (2016) [22]	Spain	Single-center retrospective study	Comparison of clinical features, treatment strategies, and outcomes between T2MI and T1MI	117 patients	Obstructive CAD 33%	Obstructive CAD is more common among T1MI patients
Saaby (2013) [8] (DEF-AMI)	Denmark	Single-center cross-sectional study	Investigate the frequency and features of T2MI	144 patients	Signifiant CAD: 54.8%	Approximately half of patients with T2MI have significant CAD
Ambrose (2012) [23]	USA	Single-center cross-sectional study	Severity of CAD between T2MI and T1MI	31 patients	Previous CAD: 45.2% 3-vessel CAD: 32.3% (T2MI) vs. 26.6% (T1 NSTEMI) and 4.1% (T1 STEMI)	T2MI patients with significant CAD appear to be more severe with more 3-vessel disease compared to patients with T1MI

CAD: coronary artery disease. T2MI: type 2 myocardial infarction. T1MI: type 1 myocardial infarction. STEMI: ST-segment elevation myocardial infarction. NSTEMI: non-ST-segment elevation myocardial infarction. HR: hazard ratio. CI: confidence interval. CRP: C-reactive protein. DEMAND-MI: Determining the Mechanism of Myocardial Injury and Role of Coronary Disease in Type 2 Myocardial Infarction. High-STEACS: High-Sensitivity Troponin in the Evaluation of patients with Acute Coronary Syndrome. SWEDEHEART: Swedish Web-system for Enhancement and Development of Evidence-based care in Heart disease Evaluated According to Recommended Therapies. RICO: ObseRvatoire des Infarctus de la Côte d’Or. CASABLANCA: Catheter Sampled Blood Archive in Cardiovascular Diseases. BACC: Biomarkers in Acute Cardiac Care. AMIS PLUS: national Registry of Acute Myocardial Infarction in Switzerland. DEF-AMI: consequences of the universal 2007 DEFinition of Acute Myocardial Infarction studied in a Danish consecutive hospital population.

**Table 2:** Definition criteria of acute stressors in type 2 myocardial infarction.

Mechanism	Definition
Sustained tachy-arrythmia [8]	supraventricular tachyarrhythmia ≥ 20 min with a ventricular rate >150 beats/min
Severe hypertension [8]	systolic blood pressure > 160 mmHg, with or without concomitant LVH identified by echocardiography
Severe bradyarrhythmia [8]	bradyarrhythmia requiring medical treatment or pacing
Respiratory failure [9]	clinical signs of acute respiratory distress lasting ≥ 20 min and arterial oxygen tension < 8kPa
Severe anaemia [8]	hemoglobin concentration < 5.5 mmol/L for men and < 5.0 mmol/L for women (measured on admission) and/or the need to use blood products
Hypotension/shock [25]	systolic BP < 90 mmHg and/or diastolic BP < 60 mmHg is associated with evidence of systemic hypo-perfusion (e.g., hyperlactatemia) and low cardiac output.
Spontaneous coronary artery dissection [7]	was defined as spontaneous dissection of the coronary artery wall with accumulation of blood within the false lumen, which can compress the true lumen to varying degrees
Coronary spasm [4]	refers to a sudden, intense vasoconstriction of an epicardial coronary artery that causes vessel occlusion or near occlusion on coronary angiography, even in the absence of stimulation
Coronary embolism [8]	defined as a high thrombus burden despite a relatively normal underlying vessel or recurrent coronary thrombus (left heart endocarditis, intracardiac mural thrombus, documented venous thrombus, and a patent foramen ovale or atrial septum defect)

LVH: left ventricular hypertrophy. BP: blood pressure.

**Table 3:** Prognosis of T2MI patients with CAD.

First Author (Date)	Follow-Up	T2MI Patients (CAD/no CAD)	Endpoint	Death Rate (T2MI CAD/T1MI CAD/T2MI without CAD)	Crude HR [95%CI] (T2MI CAD vs. T2MI without CAD)	Crude HR [95%CI] (T2MI CAD vs. T1MI)	Adjusted HR [95%CI] (T2MI CAD vs. T1MI)
Putot (2019) [17] (RICO)	In-hospital	254/619	All-cause death	15.0/6.6/..	..	..	..
Baron (2016) [12] (SWEDEHEART)	1.9 years	695/621	All-cause death	..	..	1.72 [1.45–2.03]	0.76 [0.61–0.94] *
Chapman (2018) [11]	4.9 years	325/467 **	MACE ***	..	1.71 [1.31–2.24]	1.56 [1.29–1.88]	..

T2MI: type 2 myocardial infarction. T1MI: type 1 myocardial infarction. CAD: coronary artery disease. RICO: ObseRvatoire des Infarctus de la Côte d’Or. SWEDEHEART: Swedish Web-system for Enhancement and Development of Evidence-based care in Heart disease Evaluated According to Recommended Therapies. MACE: major adverse cardiovascular events; * adjustment for age, sex, comorbidities, treatments, triggering mechanisms, and troponin concentration; ** T2MI or myocardial injury; *** defined as cardiovascular death or subsequent myocardial infarction.

## Data Availability

The data presented in this study are available on request from the corresponding author.
